# Mantle cell lymphoma negative for t(11,14) involving the kidneys: a case report

**DOI:** 10.1186/s13256-022-03470-z

**Published:** 2022-06-30

**Authors:** Hasan Nassereldine, Razan Mohty, Hussein Awada, Iman Abou Dalle, Jean El-Cheikh, Ali Bazarbachi

**Affiliations:** 1 Faculty of Medicine, American University of Beirut, Beirut, Lebanon; 2 Division of Hematology and Oncology, Department of Internal Medicine, American University of Beirut Medical Center, P.O. Box 113-6044, Beirut, Lebanon

**Keywords:** Non-Hodgkin’s lymphoma, Renal manifestation, Hematological malignancy, Renal failure, Case report

## Abstract

**Background:**

Mantle cell lymphoma is the rarest subtype of non-Hodgkin’s lymphoma. It can exhibit diverse extranodal manifestations. However, renal involvement is uncommon, and if it occurs, it usually only gets detected postmortem. There are several mechanisms by which mantle cell lymphoma can damage the kidneys. Renal failure is a potential complication, and prompt evaluation and diagnosis are critical steps to prevent long-term complications.

**Case presentation:**

We present the case of a 75-year-old non-Hispanic White male with past medical history significant for hypertension and dyslipidemia, presenting with fever, weight loss, and night sweats. Work-up showed markedly elevated white blood cells, multiple enlarged lymph nodes, and a kidney mass. The patient was diagnosed with mantle cell lymphoma with kidney involvement confirmed with a kidney biopsy. His disease was positive for cyclin D1 overexpression despite t(11; 14) absence. The patient received six cycles of alternating vincristine, rituximab, cyclophosphamide, doxorubicin, and prednisone then dexamethasone, high-dose cytarabine, and oxaliplatin, after which he was maintained on ibrutinib and rituximab, with resolution of symptoms and disease regression.

**Conclusion:**

We present a case of a rare presentation of Mantle cell lymphoma while describing the clinical presentation and diagnostic and treatment approaches. This case report can assist physicians in the clinical work-up and treatment of patients with similar diagnosis or presentation.

## Background

Mantle cell lymphoma (MCL) is a rare subtype of B-cell non-Hodgkin lymphoma (NHL) that accounts for 2.5–6% of all NHL. It is characterized by the propagation of monoclonal lineage of mature B-lymphocytes [1] and associated with t(11;14)(q13;q32) gene translocation leading to overexpression of the cell-cycle regulatory protein cyclin D1 [2]. MCL is a disease of the older population with median age at onset of 60 years [3–5]. The median overall survival (OS) of these patients is around 3–5 years [6, 7]. Approximately 80% of patients have advanced stage at diagnosis, with the most common sites of extranodal involvement being the bone marrow, liver, spleen, Waldeyer’s ring, and gastrointestinal tract [3, 8, 9]. Conversely, kidney involvement is very rare and is often underdiagnosed. However, the increase in the incidence and survival of those patients, in the last few decades, has allowed for such uncommon manifestations to become more apparent [10]. The mechanism by which the disease can affect the kidneys are various and might be due to direct infiltration, urinary tract obstruction, or as a consequence of paraneoplastic syndrome, leading to a wide differential of presentations including acute kidney injury, nephrotic syndrome, glomerulopathy, or acute tubule-interstitial nephritis [10, 11]. In this paper, we describe a case of an elderly patient with cyclin D1-positive, t(11;14)-negative MCL with kidney involvement at diagnosis.

## Case presentation

A 75-year-old non-Hispanic White male with past medical history significant for hypertension and dyslipidemia presented with 1-month history of flu-like illness characterized by fever reaching 38.5 °C, nonproductive cough, weight loss, and occasional night sweats. The patient denied any skin rashes, change in bowel movements, urinary symptoms, or joint pain. The patient is a nonsmoker with no history of alcohol intake and additionally reports no family history of malignancies. Physical examination on presentation was pertinent for diffuse wheezing and rhonchi in addition to abdominal distention and bilateral axillary lymphadenopathy; the patient had no apparent skin changes. The patient had no financial difficulties and was addressed with his preferred native language of Arabic. Upon admission, his complete blood count (CBC) showed elevated white blood cell (WBC) count (18,400/μL) and low hemoglobin (Hb) (6.4 g/dL), for which he received 2 units of packed red blood cells (pRBCs). Notably, he was found to have an elevated creatinine level (5.9 reaching 6.5 mg/dL) and low albumin (2.8 g/dL). Hemolytic work-up was negative (Table 1).

**Table 1:** Summary of relevant laboratory results

Laboratory test	Result	Reference range
WBC	18,400/μL	4000–11,000/μL
Hemoglobin	6.4 g/dL	12.0–18.0 g/dL
Creatinine level	5.9-6.5 mg/dL	0.5–1.0 mg/dL
Albumin level	2.8 g/dL	36–53 g/L
Beta-2 microglobulin	8.98 mcg/mL	0.80–2.40 mg/L
Direct Coombs test	Negative	Negative
Reticulocyte count	1.25%	0.2–2.0%
Indirect bilirubin	0.1 mg/dL	0.2–0.8 mg/dL
Haptoglobin	2.69 mg/dL	0.30–2.00 g/L
LDH	209 IU/L	110–265 IU/L
Peripheral smear	No schistocytes or spherocytes; no abnormal cells	

*WBC* white blood count, *LDH* lactate dehydrogenase

**Fig. 1:**
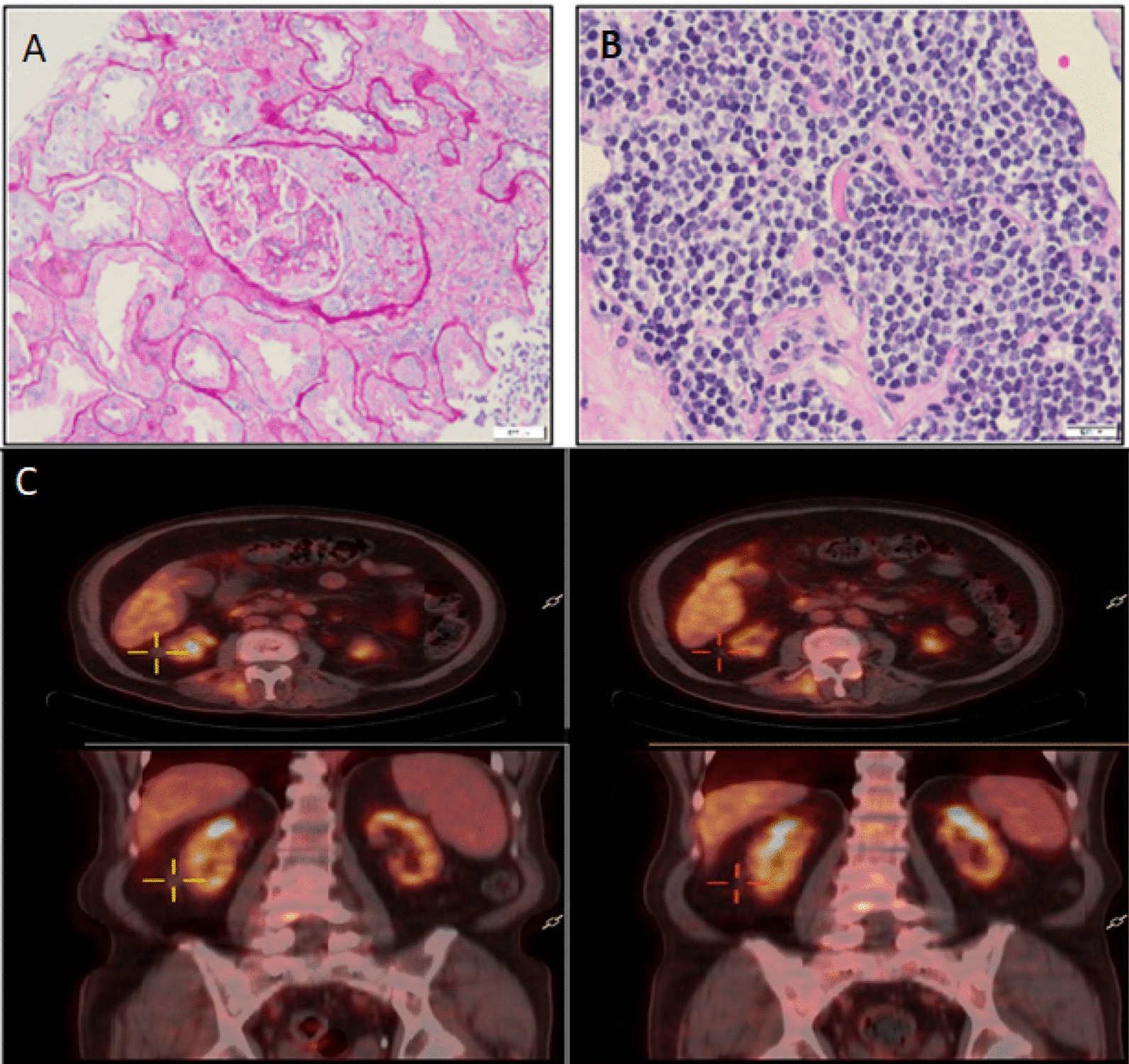
Kidney biopsy results: **A** Focal tubular atrophy and a glomerulus with cellular crescent are noted **B** The interstitial compartment shows, in addition to the inflammatory cell infiltrate, aggregates of monomorphic neoplastic lymphoid cells. PET-CT scan **C** on the left image showing a mass on the lower pole of the left kidney (yellow crosshair). Right image of PET-CT scan shows resolution of the mass after 3 months of chemotherapy

A computed tomography (CT) scan showed features of pneumonia, bilateral abnormal axillary lymph nodes (LN), splenomegaly (22 cm), and scattered abnormal LN across the abdomen and pelvis. Ultrasound (US)-guided axillary LN core biopsy revealed cells positive for CD5, CD20, CD 23, and cyclin D1 but negative for CD3 and CD10, with Ki-67 of 15% suggestive of MCL. Positron emission tomography (PET) scan showed fluorodeoxyglucose (FDG) avid supra- and infradiaphragmatic lymphadenopathy as well as splenic and lung involvement as well non-FDG-avid outer parenchymal right kidney mass (Fig 1C). Bone marrow biopsy was performed, showing disease involvement. Molecular studies for t(11;14) were negative, and cytogenetic studies showed normal karyotype. His MCL international prognostic index (MIPI) was high (6.8).

Work-up for elevated creatinine was pursued. US of the kidney showed increase in cortical echogenicity with normal thickness, denoting renal parenchymal disease. Serum protein electrophoresis and immunofixation showed protein bands of restricted mobility in the gamma region, corresponding to a monoclonal IgG-lambda as part of an oligoclonal pattern of IgG kappa and lambda. Urine analysis was positive for proteins, Hb, and numerous RBCs. Due to the unclear etiology of renal disease, CT-guided kidney biopsy was done, revealing mesangial hypercellularity, tubular atrophy, interstitial fibrosis, focal chronic inflammation with few plasma cells, and atypical lymphoid infiltrate positive for CD20 and cyclin D1. Immunofluorescence study showed diffuse mesangial positivity for IgA (Fig 1A, B). Such findings were consistent with acute tubular injury and acute interstitial nephritis due to renal involvement by a known MCL.

**Fig. 2:**
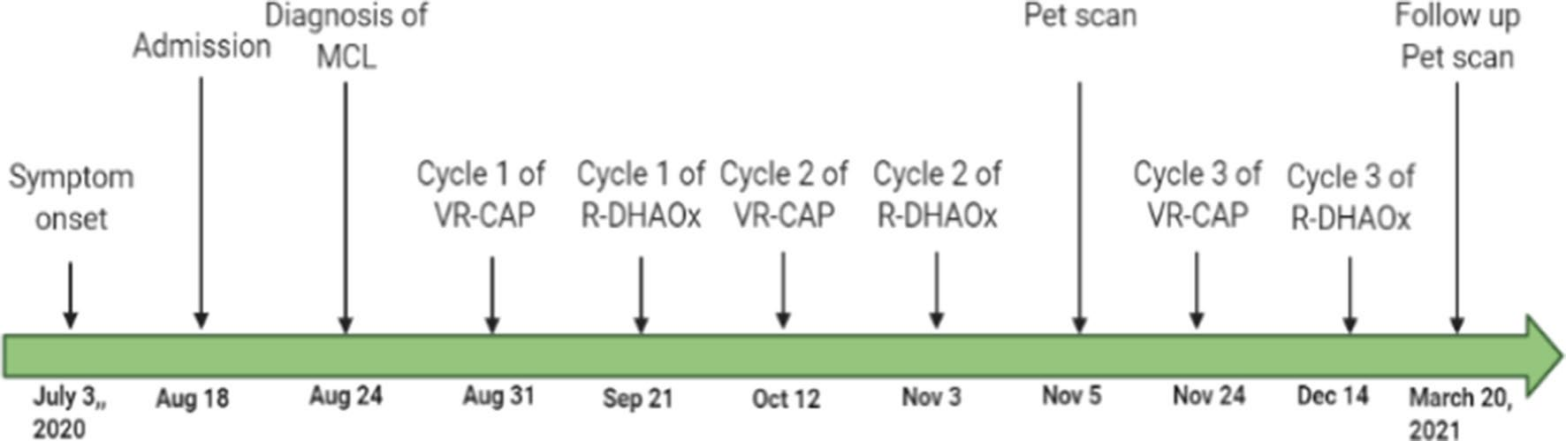
Timeline of symptom onset, patient diagnosis, and treatment

The patient received six cycles of alternating vincristine, rituximab, cyclophosphamide, doxorubicin, and prednisone (VR-CAP) and rituximab, dexamethasone, high-dose cytarabine, and oxaliplatin (R-DHAOx). His treatment was complicated by recurrent admissions for pneumonia and febrile neutropenia. Disease evaluation after four cycles of treatment by PET scan showed resolution of supra- and infradiaphragmatic disease with a Deauville score of 1 (Fig 1C). End-of-treatment evaluation showed no FDG-avid disease, with complete resolution of the kidney mass with uptake in the upper poles of the kidney. Follow-up studies showed improvement in kidney function as evidenced by a creatinine level now ranging between 3.1 and 3.6 mg/dL. He was started on rituximab maintenance. After 3 months of follow-up, PET scan showed increased uptake in the kidneys without another lymphadenopathy. He was started on ibrutinib, a Bruton tyrosine kinase inhibitor, in addition to continuation of rituximab. PET scan after 3 months of ibrutinib with rituximab showed complete remission with a Deauville score of 3. A timeline of the patient’s disease course is presented in Fig 2.

## Discussion

Our patient had dual acute tubular injury and interstitial nephritis secondary to lymphomatous renal parenchymal invasion. Lymphoid infiltration is one of the main pathological mechanisms by which NHL, particularly MCL, directly affects kidneys, and it is often asymptomatic [12]. In one of the largest case series of autopsies conducted on lymphoma patients, it was found that 34% of patients had renal lymphoid infiltration, of which only 14% were detected before death [13]. Indeed, renal failure occurs in only 0.5% of these patients, and most of them do not develop signs of volume overload or flank pain, which explains its underdiagnosis [12]. Renal failure is thought to develop because of increased pressure within the parenchyma that results from lymphocyte invasion, as shown in our patient. This hypothesis is supported by studies underlining the concomitant improvement in renal function and decrease in kidney size after starting chemotherapy, though in most patients renal function fails to recover back to baseline [14]. Correspondingly, our patient’s renal function began to improve after starting treatment of MCL, and his serum creatinine level stabilized at a range higher than his baseline, which could be explained by the multifactorial aspects of his renal failure. The presence of a chronic element of kidney disease with 40% fibrosis on the biopsy will obviously remain as a cause of his chronic kidney disease. Also, the presence of paraproteinemia and long-standing hypertension could in part participate in his renal failure. Hence, controlling his blood pressure is essential to achieve long-term kidney function control. Although kidney function starts to recover gradually once MCL treatment is initiated, patients with such overt renal failure require immediate and focused kidney management. In addition to IV hydration, administration of immunosuppressive therapy and corticosteroids allows for rapid improvement in renal function. Nevertheless, temporary renal replacement therapy may still be necessary in some cases while adequate renal function is being restored [15, 16].

Although the presence of t(11; 14) is characteristic of MCL diagnosis, it is not a necessary finding for establishing the disease diagnosis [17]. Indeed, several reports and studies have provided evidence of MCL overexpressing cyclin D1 in the absence of t(11;14) [17, 18]. Our patient’s lymph node biopsy was positive for CD5, CD20, CD23, and cyclin D1 on flow cytometry. The finding of cyclin D1 asserts the diagnosis of MCL. While the overexpression of this marker is associated with t(11,14), evidence in literature suggests that this is not always the case [19, 20]. Although CD23 is not a common finding in MCL, it is still one of the frequently described immunophenotypic variants of MCL [21].

## Conclusion

Finally, although rare, renal involvement by MCL should always be included in the differential diagnosis when a patient with MCL presents with renal function impairment. Imaging can be very useful; however, the diagnosis might necessitate kidney biopsy for confirmation in some cases. Prompt evaluation and treatment are important to prevent long-term sequelae. Clinicians should focus on preservation of kidney function through treatment of MCL first, then focus on exacerbating factors including blood pressure and blood sugar control, and avoidance of nephrotoxins. Kidney function recovers, albeit partially, once MCL treatment is initiated.

## Data Availability

Not applicable.
